# Molecular Identification of Extrapulmonary Vaccine Adverse Events after BCG in Paraffin-Embedded Specimens

**DOI:** 10.3390/pathogens12121374

**Published:** 2023-11-21

**Authors:** Sylwia Brzezińska, Anna Zabost, Dagmara Borkowska-Tatar, Magdalena Klatt, Jolanta Goździk, Agnieszka Dłużniewska, Katarzyna Błasińska, Ewa Augustynowicz-Kopeć

**Affiliations:** 1 Department of Microbiology, National Tuberculosis and Lung Diseases Research Institute, 01-138 Warsaw, Polandd.borkowska@igichp.edu.pl (D.B.-T.);; 2 Department of Clinical Immunology and Transplantation, Jagiellonian University Medical College, 31-008 Krakow, Poland; 3 Transplantation Centre, University Children’s Hospital, 30-663 Krakow, Poland; 4 Department of Radiology, National Tuberculosis and Lung Diseases Research Institute, 01-138 Warsaw, Poland; k.blasinska@igichp.edu.pl

**Keywords:** *Mycobacterium bovis* BCG, genetic test, granulomas, histopathological specimens

## Abstract

According to the World Health Organization (WHO), around 1 million children worldwide are diagnosed with tuberculosis each year. The Bacillus Calmette–Guérin (BCG) vaccine has been used around the world for over 100 years. The complications of the BCG vaccination can occur in about 0,06% of children and include local or systemic adverse reactions. Due to the close analogy between the vaccine strain and other species of the *Mycobacterium tuberculosis* complex (MTBC), molecular methods are recommended for differential diagnosis of Vaccine adverse events (VAE) after BCG. The ability to quickly and specifically identify BCG is important in view of different treatment regimens. The aim of the study was to assess the usefulness of genetic testing for *Mycobacterium bovis* BCG in the paraffin-embedded specimens’ methods. We describe two cases of VAE in immune-compromised children presenting with osteoarticular changes that had been clinically suspected of tuberculosis and led to molecular identification through GeneXpert, GenoType MTBC, and Spoligotyping. Results: *Mycobacterium bovis* BCG was detected in osteoarticular changes embedded in paraffin block of two patients. Conclusion: Genetic tests using paraffin-embedded materials allow for quick identification and differential diagnosis of patients with Tuberculosis and VAE after BCG. This is an important issue, especially in cases where the tissue has only been submitted for histopathological examination without microbiological diagnostics for tuberculosis.

## 1. Introduction

In many countries, both developed and developing, tuberculosis in children remains a health problem that is closely related to the prevalence of the disease in adults. According to the World Health Organization (WHO), around 1 million children worldwide are diagnosed with tuberculosis each year, of whom only 400,000 are registered in national registries [1].The lack of reports involves mainly children <5 years of age. The gap in the detection of TB cases in this age group is >70%, whereas, in the age group of 5–14 years, it is about 50%. The BCG is the only antituberculosis vaccine and was introduced in Poland in 1926 [2]. The standard practice is to administer the BCG vaccine to all newborns on the first day of life. In 2022, BCG vaccination was performed in 90.1% of all liveborn children in Poland. In most cases, this practice is generally considered safe, and rare complications can occur in about 0.06% of children. In 2021, one case of severe postvaccination reaction was reported [3]. The BCG vaccine which is a live attenuated strain of *Mycobacterium bovis* is safe and has a well-documented protective effect against meningitis and disseminated tuberculosis in young children [4]. The complications of BCG vaccination have been debated for a long time and include local side effects such as injection site abscesses, ulceration, keloid reactions, as well as systemic side effects such as osteomyelitis. Most lymphadenopathies associated with BCG complications resolve spontaneously in immunocompetent patients, whereas severe disseminated BCG infection can occur in children with primary immunodeficiency. A lot of research indicates that a coexisting HIV infection may result in an adverse post-vaccination reaction in the form of a life-threatening disseminated *M. bovis* BCG infection [5]. Unfortunately, a diagnosis of immunodeficiency is very often diagnosed only after BCG complications have occurred [6]. Quick and sensitive diagnosis of childhood tuberculosis is the key to limiting the development of the disease and preventing its transmission. Diagnosis of the disease is difficult due to the mild clinical course, non-specific symptoms, and difficulties in obtaining bacteriological confirmation. These difficulties result from the nature of pulmonary tuberculosis (PTB) and extrapulmonary tuberculosis (EPTB), with a low count of mycobacteria. Diagnosis is further complicated by the difficulties in obtaining the appropriate amount of material, as young children cannot effectively expectorate sputum. Therefore, the material for microbiological diagnostics obtained from children should be treated as unique, and all available diagnostic methods should be used in this group of patients. The diagnosis of pulmonary tuberculosis in a child, without bacteriological confirmation, must be based on a thorough medical history, physical examination, the results of a skin test (TST) and/or an IGRA test, and chest X-ray [7]. As most of these examinations have low specificity, we should aim at increasing the percentage of tuberculosis cases confirmed bacteriologically by modern microbiological diagnostic methods. In the case of failure to perform microbiological diagnostics for tuberculosis and fixation of all the material in formalin, the only chance to confirm the presence of *M. tuberculosis* complex is to perform a genetic test using a pathomorphological specimen. 

The diagnosis of *M. bovis* BCG infection is based on vaccination history, clinical signs and symptoms, and the results of microbiological tests. Conventional mycobacterial identification methods do not distinguish the BCG strain from other species within the MTBC. Consequently, in some cases of disseminated *M. bovis* BCG infection, the disease may be registered and treated as tuberculosis. Currently, molecular testing allows for rapid identification of the *M. bovis* BCG vaccine strain and initiation of proper treatment. It is very important in the case of BCG-induced side effects, such as osteitis, that the disease often begins with insidious, non-specific symptoms that lead to delayed clinical diagnosis, and this can result in extensive damage and serious complications. 

## 2. Methods

For materials subjected to genetic testing several stages of diagnostics were performed:

### 2.1. Pathomorphological Examination

Preparation of tissue material for pathomorphological examination (fixation, dehydration, and paraffin embedding of the material) and slide staining (Hematoxylin + Eosin H + E, Ziehl-Neelsen Z-N) [8].

### 2.2. Genetic Analysis

○ The formalin-fixed, paraffin-embedded tissues were deparaffinised and then lysed (with proteinase K). DNA was extracted in an automated system Maxwell Promega (16 FFPE Plus Lev DNA Purification Kit);
○ The genetic material was then analysed by: Xpert MTB/RIF Ultra (Cepheid https://www.cepheid.com/content/dam/www-cepheid-com/documents/package-insert-files/Xpert-MTB-RIF-ENGLISH-Package-Insert-301-1404-Rev-G.pdf, accessed on 1 July 2020—detection of specific sequences to MTBC;
○ GenoType MTBC;
○ Spoliogtyping (Spoligotyping Kit Manual, Isogen Life Science)—identification of *Mycobacterium bovis* BCG.

### 2.3. Culturing Methods

Specimens were processed—decontamination of material with N-acetyl-L-cysteine–sodium hydroxide—and inoculated into the BACTEC MGIT 960 Middlebrook 7H9 (liquid culture) and onto Lowenstein-Jensen L-J (solid culture) [9].

## 3. Case I

A child with no family history of immunodeficiency received the BCG vaccine on the second day of life. Abnormal physical development and recurrent lower respiratory tract infections have been observed since infancy. The child was diagnosed with severe combined immunodeficiency: T cell-negative, B cell-positive, and natural killer cell-negative (T-B + NK-SCID). Hematopoietic cell transplantation was performed twice: at the age of 10 months with full ablation preparation and at the age of 13 months without conditioning. The patient’s immune reconstitution was significantly delayed—13 months after the first transplantation, the level of CD4 cells was 200/µL; 22 months after stem cell transplantation SCT, this level normalized. After transplantation, exacerbation of respiratory symptoms with increasing dyspnoea, diffused infiltrative changes with features of fibrosis in the chest tomography, and the interstitial process in the lung ultrasound were observed. Inflammation of the intestines was also reported. The presence of *Candida* spp. was confirmed in BAL and *Candida glabrata* in the stool. The isolates were cultured and identified by BBL™ CHROMagar Candida (Becton, Dickinson and Company, Sparks Glencoe, MD, USA) and identified by System API Candida (bioMérieux S.A, Marcy-l’Étoile, France).

**Figure 1:**
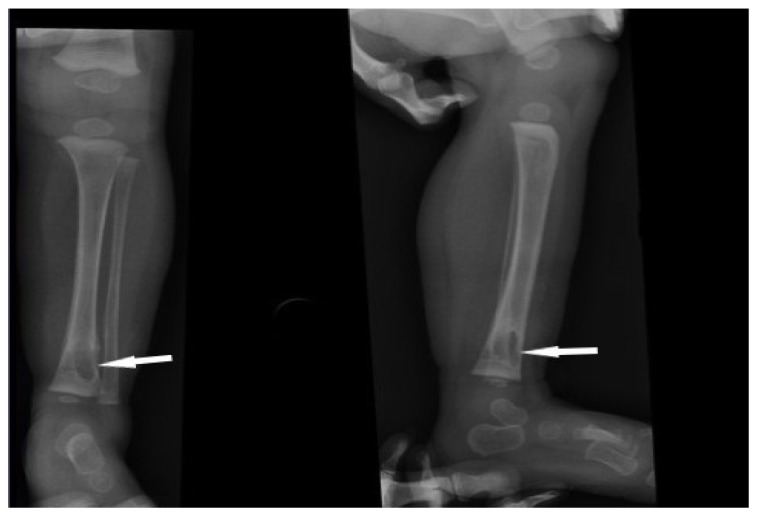
Radiographic image of bone osteolytic changes within the distal metaphysis of the tibia (white arrows)—at the time of diagnosis.

The introduction of antifungal treatment and modified antibiotic therapy resulted in regression of the acute inflammatory process in the lungs and improvement of respiratory efficiency. At the age of 2 years, an induration and infiltrative tumour in the left lower leg were found due to partial reconstitutions of immunity. Medical imaging (Computed tomography, CT; Magnetic resonance imaging, MRI) revealed a mass within the distal femoral metaphysis and a solid infiltrative lesion within the diaphysis and distal metaphysis of the tibia. Radiographic image (Radioisotope thermoelectric generator, RTG) showed the destructive changes within the distal metaphysis of the tibia (Figure 1).

The histopathological examination of the left lower leg tumour showed the presence of granulation tissue containing macrophages and multinucleated giant cells with necrotic and purulent infiltrates. Z-N and PAS staining did not reveal any mycobacteria or fungi. Based on the result of histopathological examination and clinical observations, the material from the paraffin block was sent for molecular diagnostics. The presence of *Mycobacterium tuberculosis* complex DNA was detected (Xpert MTB/RIF Ultra test). The GenoType MTBC molecular test was used to identify the species of *Mycobacterium bovis* BCG. 

**Figure 2:**
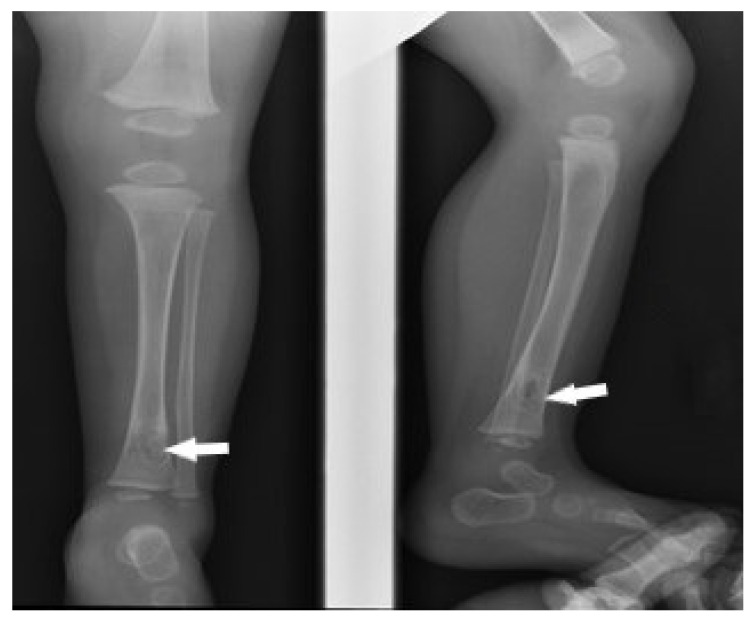
Radiographic image of changes in the distal metaphysis of the tibia (white arrows)—after 3 months of treatment. Partial but significant regression of bone lesions.

Based on the result, the appropriate antimycobacterial therapy (rifampicin, isoniazid, ethambutol, and levofloxacin) was introduced, excluding pyrazinamide, to which *M. bovis* BCG is naturally resistant. The targeted treatment led to a significant improvement in the imaging examination (Figure 2). The patient in good condition remained under observation.

## 4. Case II

**Figure 3:**
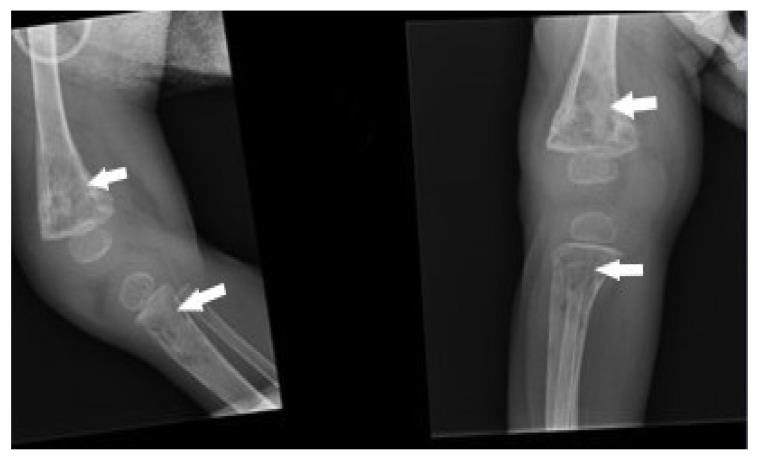
Radiographic image of bone destructive changes in distal metaphysis of the femur and proximal metaphysis of the tibia (white arrows).

A patient with no history of immunodeficiency was vaccinated with the BCG vaccine according to the current vaccination calendar (on the first day of life). From early infancy, impaired physical development, recurrent thrush, and disseminated skin abscesses in many sites were observed. An abnormal formation of the scar after BCG vaccination, oozing inflammatory infiltration, and single subcutaneous nodules at the site of inoculation were also reported. The diagnosis of severe combined immunodeficiency: T cell-negative, B cell-positive, natural killer-negative (T-B + NK-SCID) were made, and hematopoietic cell transplantation (from brother) was performed at the age of 10 months without conditioning. Due to chronic pneumonia, bronchoscopy was performed on the 4th day after transplantation. Testing *Bocavirus* (polymerase chain reaction PCR-NP1 protein), *Candida* spp. (BBL™ CHROMagar Candida Becton Dickinson), and *M. tuberculosis* (Middlebrook 7H9 medium, Stonebrink and Löwenstein–Jensen medium) in the BAL was negative. Despite the treatment, the febrile condition persisted, and the features of liver, spleen, and kidney failure were also noted. Steroid therapy and immunosuppressive treatment were started, which resulted in improvement of the child’s general condition. RTG was performed at the age of 11 months, and osteolytic foci in the right femur and tibia were found (Figure 3).

**Figure 4:**
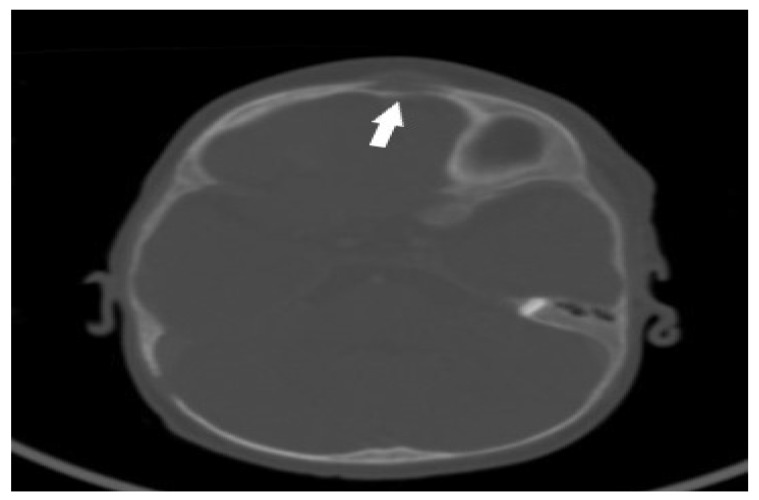
CT image of the head—frontal osteolytic bone lesion with soft tissue infiltration (white arrow).

Imaging studies showed osteolytic changes with features of granulation tissue proliferation in the frontal bone and changes in the temporal region. In addition, inflammatory subcutaneous nodules were noted in the soft tissues of the forehead, third rib, and left humerus. A CT of the head (Figure 4) showed frontal bone loss with soft tissue infiltration at the time of diagnosis.

**Figure 5:**
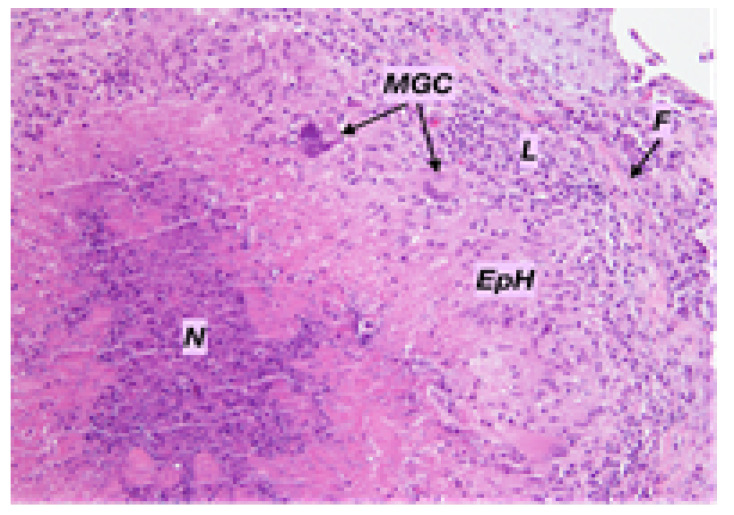
Microscopic appearance of necrotizing tuberculoid granuloma. Hematoxylin and Eosin staining, magnification (×200). N = necrosis, EpH = epithelioid histiocytes, MGC = multinucleated giant cells, L = lymphocytes, and F = fibrotic tissue.

A multi-stage diagnosis of tuberculosis was made based on an examination of the tumour from the frontal region and an epidermal cyst from the temporal region. The histopathological specimen confirmed the presence of granulomas with necrosis, multinucleated giant cells, and fibrotic tissue (Figure 5). 

**Figure 6:**
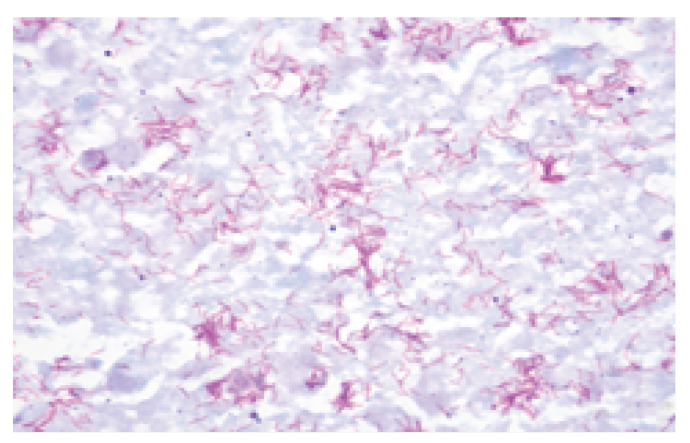
Acid-fast bacilli in the histological specimen (Z-N staining).

Microscopic examination (Z-N staining) confirmed the presence of acid-fast bacilli (Figure 6).

Genetic testing of the material from the paraffin block confirmed the presence of *Mycobacterium tuberculosis* complex (MTBC) DNA, which allowed for the rapid initiation of antimycobacterial treatment. Microscopic examination (Z-N staining) of the liver biopsy confirmed the presence of *Mycobacterium*. Microbiological diagnostics of the lymph nodes revealed the presence of mycobacteria (L-J medium). Identification the strain by spoligotyping revealed the presence of *M. bovis* BCG. Treatment was administered (rifampicin, isoniazid, streptomycin, and levofloxacin), with gradual resolution of the local and systemic infection. After a year, a splenectomy was performed due to the presence of a cyst in the spleen and the presence of MTBC genetic material was confirmed, and no mycobacterial culture was obtained.

The boy developed chronic liver damage and failure of the mixed aetiology covering immunological (GvHD), infectious (tuberculosis), and toxic (drugs) factors. The patient was qualified for family liver transplantation. The clinical condition after the transplantation was good; however, the patient required substitution of immunoglobulins and remained under observation.

## 5. Discussion

The clinical cases described in the paper show the need to use all methods available to diagnose MTBC, especially in children, in whom non-specific symptoms significantly hinder the making of a final diagnosis. The BCG vaccine is one of the safest live bacterial vaccines; however, the immunization process can lead to local and systemic adverse events [4]. Osteitis/osteomyelitis after BCG vaccination, although considered rare in the population, has been reported in studies with an incidence rate ranging from 0.01 to 300 per 1,000,000 [10,11]. In patients with a properly functioning immune system, generalized *M. bovis* BCG infection is extremely rare. While prognosis for BCG lymphadenitis is good, in patients with disseminated BCG infection, the outcome is often poor [12]. Due to the lack of characteristic symptoms of disseminated BCG, confirming the etiological factor of the disease is challenging. This is particularly important when deciding on the treatment of mycobacterial infection, considering *M. bovis* BCG’s natural resistance to pyrazinamide (PZA), one of the main antituberculosis drugs. Therefore, in cases of suspected BCG infection, detailed tests differentiating species within the MTBC are essential. Clinical suspicion of VAE after BCG vaccination and congenital or acquired immune system dysfunctions are absolute indications for such identifications. Of particular attention is the severe disseminated generalized form after BCG vaccination, which usually occurs in patients with immunological disorders such as severe combined immunodeficiency, IFN-γ or Il-12 receptor defects, or chronic granulomatous disease [5]. Radiological reports on disseminated BCG infection typically describe changes suggestive of osteomyelitis [13]. In adults, the infection caused by the vaccine strain can occur after the treatment of bladder cancer with intravesical BCG instillations. The therapy is generally safe and well tolerated, although some side effects may appear, both local (cystitis and prostatitis) and systemic (fever, tuberculous spondylitis, and granulomatous hepatitis) [14]. Pulmonary *M. bovis* BCG infection and sepsis are very rare, occurring in only 0.4% of patients [15]. While among the most frequently diagnosed extrapulmonary tuberculosis (EPTB) forms are tuberculous lymphadenitis, gastrointestinal tuberculosis, and osteoarticular tuberculosis [16], the incidence of osteitis/osteomyelitis is estimated to be around 10–13% of EPTB cases and 1–5% of all tuberculosis cases [17,18]. The late diagnosed and untreated osteoarticular form of TB can lead to serious complications and can be a source of functional disability. Therefore, early diagnosis and treatment is especially important in children, in whom appropriate therapy can bring about full recovery.

The osteoarticular form of *M. bovis* BCG infection is most commonly diagnosed in children, usually to two years after vaccination, and develops insidiously due to the slow progression of lesions. Clinical and imaging findings were retrospectively evaluated in 14 children with BCG osteomyelitis, including three with Mendelian susceptibility to mycobacterial diseases (MSMD). Immunocompetent patients came to medical attention on average 14 months after vaccination, whereas patients with MSMD came much earlier (average 5 months). The former manifested with a slowly progressive, painless mass with only mildly increased acute-phase reactants, whereas the latter started with lymphadenitis with significant inflammatory reactions and later developed osteomyelitis [19]. Non-specific clinical symptoms significantly hinder the final diagnosis of *M. bovis* BCG infection. In the absence of suspicion of *Mycobacterium* infection, the material collected during surgical procedures is solely sent for histopathological examination. Histology of biopsy shows granulomatous reaction with caseous necrosis, whereas, for Ziehl–Neelsen staining to become positive, biopsy material must contain a minimum of 10,000 bacteria per gram of tissue [12]. In such situations, detection and identification of the mycobacteria to the species level are possible only based on genetic examination of the material from a paraffin block [20]. The studies on formalin-fixed, paraffin-embedded tissues indicate that the causes of false negativity might be: nucleic acid fragmentation secondary to formalin fixation and nonspecific product formation, especially when the target concentration is low, and background DNA is high [12]. Determining the species is crucial from a clinical perspective, especially considering the different treatment regimens, particularly due to *M. bovis* BCG’s natural resistance to pyrazinamide (PZA), one of the main anti-tuberculosis drugs. The prognosis for children with BCG strain bone infections largely depends on the condition of the immune system, type of immune disorder, and the possibility of its treatment, as well as on anti-mycobacterial therapy. Therefore, detailed microbiological and molecular diagnostics of the *M. tuberculosis* complex are of key importance in the further management of a patient with suspected *M. tuberculosis*/*M. bovis* BCG.

In conclusion, microbiological and molecular diagnostics of the *Mycobacterium tuberculosis* complex to the species level are crucial for further management of patients with suspected adverse events and complications after BCG vaccination.

## Data Availability

The clinical data of the patient are available in the hospital database.
